# Tree-ring oxygen isotopes record a decrease in Amazon dry season rainfall over the past 40 years

**DOI:** 10.1007/s00382-021-06046-7

**Published:** 2021-11-26

**Authors:** Bruno B. L. Cintra, Manuel Gloor, Arnoud Boom, Jochen Schöngart, Jessica C. A. Baker, Francisco W. Cruz, Santiago Clerici, Roel J. W. Brienen

**Affiliations:** 1 School of Geography, University of Leeds, Garstang North Building, Leeds, LS2 9JT UK; 2 Institute of Biosciences, University of São Paulo, Rua do Matão 14, São Paulo, 05508-090 Brazil; 3 School of Geography, Geology and the Environment, University of Leicester, Bennet Building, University Road, Leicester, LE1 7RH UK; 4 Coordination of Environmental Dynamics, National Institute for Amazon Research, Av. André Araújo 2936, Petrópolis, Manaus, 69067-375 Brazil; 5 School of Earth and Environment, University of Leeds, Leeds, UK; 6 Institute of Geosciences, University of São Paulo, Rua do Lago 562, São Paulo, 05508-080 Brazil

**Keywords:** Amazon floodplains, Climate change, Macrolobium acaciifolium, Oxygen isotopes

## Abstract

Extant climate observations suggest the dry season over large parts of the Amazon Basin has become longer and drier over recent decades. However, such possible intensification of the Amazon dry season and its underlying causes are still a matter of debate. Here we used oxygen isotope ratios in tree rings (δ^18^O_TR_) from six floodplain trees from the western Amazon to assess changes in past climate. Our analysis shows that δ^18^O_TR_ of these trees is negatively related to inter-annual variability of precipitation during the dry season over large parts of the Amazon Basin, consistent with a Rayleigh rainout model. Furthermore δ^18^O_TR_ increases by approximately 2‰ over the last four decades (~ 1970–2014) providing evidence of an Amazon drying trend independent from satellite and in situ rainfall observations. Using a Rayleigh rainout framework, we estimate basin-wide dry season rainfall to have decreased by up to 30%. The δ^18^O_TR_ record further suggests such drying trend may not be unprecedented over the past 80 years. Analysis of δ^18^O_TR_ with sea surface temperatures indicates a strong role of a warming Tropical North Atlantic Ocean in driving this long-term increase in δ^18^O_TR_ and decrease in dry season rainfall.

**Supplementary Information:**

The online version contains supplementary material available at 10.1007/s00382-021-06046-7.

## Introduction

The Amazon Basin is the world’s largest watershed and one of the wettest locations on the planet. The region drains over 6 mio. km^2^, contributing ~ 17% of the global freshwater discharge to the oceans (Richey et al. 1986; Callède et al. 2010). Most of the region’s precipitation falls during the peak monsoon months in the southern hemisphere summer (Vera et al. 2006; Garreaud et al. 2009; Marengo et al. 2012), with heavy precipitation followed by a steady rise in river levels, often leading to large-scale floods (Marengo and Espinoza 2016; Barichivich et al. 2018). In contrast, during the driest quarter of the year most of the basin undergoes a well-defined dry season, sometimes intense enough to cause severe droughts and wildfires (Tomasella et al. 2011; Aragão et al. 2018; Marengo et al. 2018). These hydroclimatic extremes impact the carbon balance and biodiversity of the largest rainforest area on the globe (Gatti et al. 2014; Esquivel-Muelbert et al. 2018; Aleixo et al. 2019), and also cause severe social and economic hardship (Marengo et al. 2013; Pinho et al. 2015; Brondízio et al. 2016).

Climate extremes in the Amazon region have become more frequent over recent decades, likely driven by changes in sea surface temperatures (SST) of the surrounding ocean basins, affecting convective activity over tropical South America and influencing the moisture inflow into the basin by changing the trade-winds from the Tropical Atlantic Ocean (Jiménez-Muñoz et al. 2016; Li et al. 2016; Barichivich et al. 2018). This has led to increases in wet season rainfall mainly in the west of the Amazon (Gloor et al. 2013, 2015), with consequent increases in frequency and magnitude of flooding extremes in Amazonian rivers (Espinoza et al. 2014; Ovando et al. 2016; Barichivich et al. 2018; Wang et al. 2018). In parallel, evidence suggests that there have been small reductions in dry season rainfall and an extension of dry season length in the south and eastern parts of the Amazon (Fu et al. 2013; Gloor et al. 2013; Marengo et al. 2018), but also possibly in more western and central regions (Ronchail et al. 2018; Gatti et al. 2021). Nonetheless, available instrumental climate data to assess the magnitude and variability of climate within this vast region are relatively scarce.

Even small reductions in dry season rainfall can exert significant influence on forest structure and biodiversity patterns (Quesada et al. 2012; Souza et al. 2016; Esquivel-Muelbert et al. 2017, 2018), by causing widespread tree mortality (Nepstad et al. 2007; Phillips et al. 2009; Brienen et al. 2015; Feldpausch et al. 2016) and increasing the occurrence of large-scale wildfires (Flores et al. 2017; Aragão et al. 2018). Resulting losses in forest area and function could potentially cause further warming and drying (Khanna et al. 2017; Wright et al. 2017; Zemp et al. 2017). However, uncertainties remain regarding how recent climate trends might be expected to unfold over the next century (Boisier et al. 2015; Fernandes et al. 2015; Baker et al. 2021). Addressing these uncertainties through climate model evaluation and development requires longer historical records with better spatial coverage across the Amazon than currently available from instrumental data.

A useful approach to improve our understanding of climate variability is to use natural archives that record oxygen isotope ratios (δ^18^O) of rainfall water (Vuille 2018). Rainfall δ^18^O is determined by the temperature of condensation and/or the rate of precipitation of rainfall (Dansgaard 1964; Araguás-Araguás et al. 2000; Schubert and Jahren 2015). This climatic signature of rainfall δ^18^O may be recorded in the isotopic composition of cellulose from tree rings (Libby et al. 1976; McCarroll and Loader 2004; van der Sleen et al. 2017), which can offer valuable proxy records for studying modern climate variability with annual or sub-annual resolution (Roden et al. 2009; Managave et al. 2011; Schollaen et al. 2013).

In the tropical climate of the Amazon Basin, rainfall δ^18^O has been shown to closely reflect a Rayleigh distillation of cloud water resulting from accumulated precipitation along airmass trajectories, also referred to as rainout upwind (Salati et al. 1979; Matsui et al. 1983; Vuille and Werner 2005). δ^18^O in tree rings (δ^18^O_TR_) from Amazon non-flooded *terra firme* forests have been shown to preserve this climatic signal from rainfall δ^18^O (Baker et al. 2015, 2016), providing historical records of large-scale precipitation amounts during the wet season, which is their main growing period (Brienen et al. 2012; Baker et al. 2018). In contrast, tree species from Amazon floodplains only grow when flood levels are low, which occurs after the main rainfall season retreats and mainly throughout the dry season (Schöngart et al. 2002; Worbes 2002). If the oxygen isotopic signal in the tree rings of these trees reflects Rayleigh distillation, then a δ^18^O_TR_ time-series obtained from floodplain trees are potentially suitable for obtaining records of historical climate conditions during the dry season at large-scale in the Amazon (Cintra et al. 2019).

Here we present a δ^18^O_TR_ record obtained from the floodplain tree species *Macrolobium acaciifolium* (Benth.) Benth. (Fabaceae) with the goal of providing an independent estimate of historical changes in dry season rainfall at the Amazon-wide scale. A prerequisite for using this approach is that δ^18^O_TR_ is primarily determined by plant source water δ^18^O, and that contributions from local climate effects on leaf water enrichment are relatively minor. Previous studies have shown that leaf water enrichment effects are larger under dry compared to humid conditions (Barbour et al. 2004; Kahmen et al. 2011; Cintra et al. 2019). Thus, trees growing in humid conditions are probably a more reliable recorder of the original source water δ^18^O variation. For this reason, we selected one of the wettest locations in the Amazon, at the western end of the Basin, to produce a 44-year long record of floodplain δ^18^O_TR_ of *M. acaciifolium* trees. As the main trade winds airstream over the Amazon follows an east–west direction, we expected the climatic signal in this δ^18^O_TR_ to result from cumulative rainout of air parcels over the larger portion of the Basin from the Atlantic Coast to the sampling site. We evaluate to what extent inter-annual variations in this δ^18^O_TR_ record may be interpreted by variations in dry season rainfall, and discuss implications of the trend in the record for recent climate changes observed in the Amazon Basin.

## Methods

### Species and sampling

**Fig. 1:**
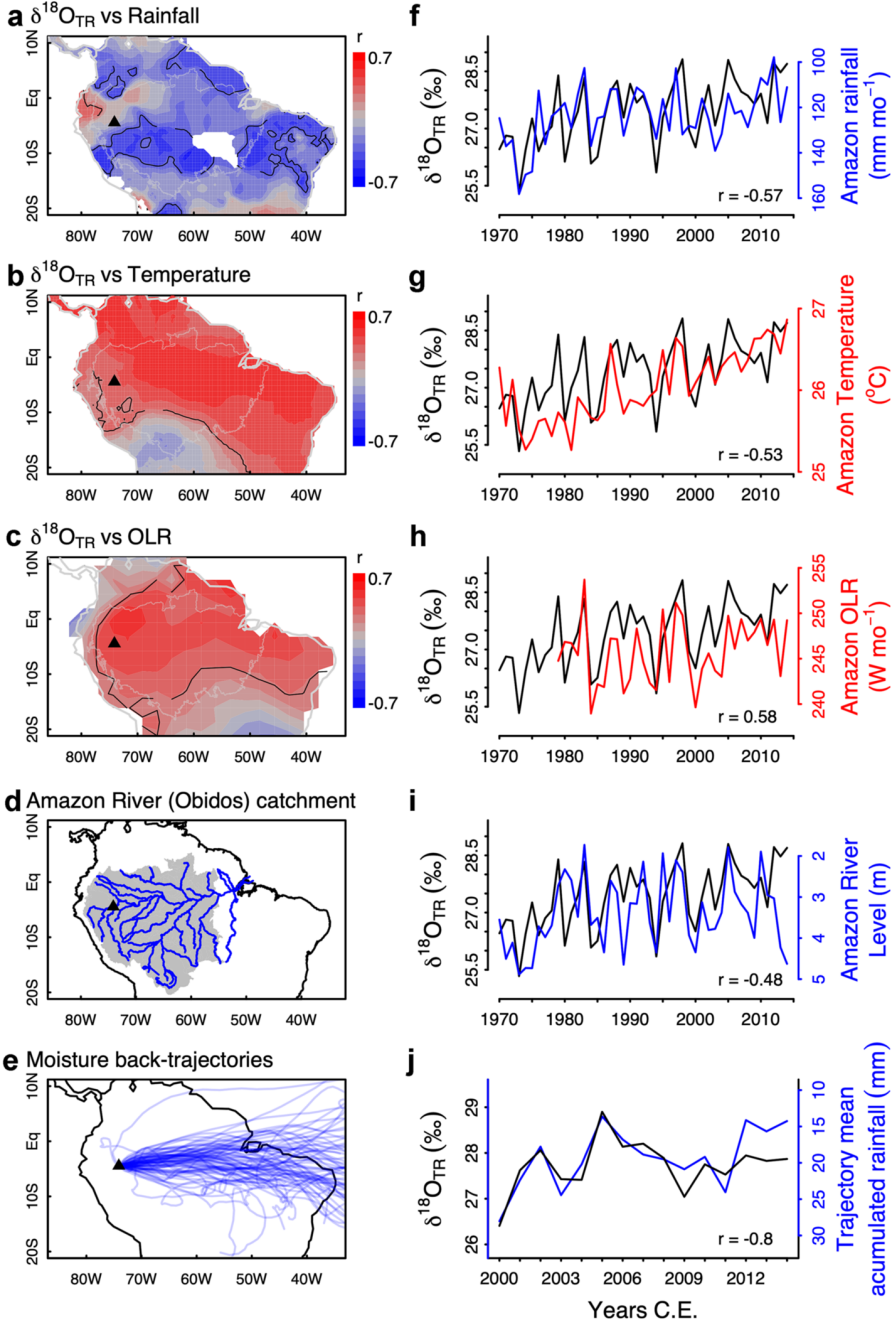
Association of the δ^18^O_TR_ record obtained from *Macrolobium acaciifolium* floodplain trees from western Peru with rainfall (**a**, **f**), temperature (**b**, **g**) from CRUTS 4.04, OLR (**c**, **h**) from NOAA, Amazon river at Obidos (**d**, **i**) from ANA/Brazil and accumulated precipitation along moisture trajectories from the sampling site (**e**, **j**) estimated from TRMM 3B42. Panels (**a**–**c**) show spatial correlation maps, with solid contours indicating regions with *p* < 0.05, and thin gray lines indicating the area used to average climate data for (**f**, **g**,** h**). Panel (**d**) shows the drainage area of the Amazon River at Obidos in grey shading and panel (**e**) shows the back-trajectories of moisture from the sampling site calculated for the height of 600 Pa. Panels (**f**–**j**) show time series comparisons of the δ^18^O_TR_ record with respective climate/river variables shown in (**a**–**e**), with blue lines indicating negative correlations shown with inverted y-axis. Amazon rainfall, temperature and OLR are shown as monthly means of dry season months from June to October. Accumulated rainfall (**i**) is shown as trajectory means from June to August. Amazon river levels are shown as monthly means from September to November. Correlation coefficients with all variables are shown in Table 1 and in Online Resource SIFig. 4

We sampled tree cores in 2015 C.E. using 10 mm increment borers at a floodplain site within the catchment of the Marañon River in Peru (74°05′30″ W, 4°29′30″ S) (Fig. 1), where rainfall normally exceeds 3000 mm year^−1^ and rarely drops below 100 mm mo^−1^. River records show an annually recurring flood-pulse (Junk et al. 1989). Due to anoxic conditions during flooding, the growth period of trees occurs during the low stage of the river (Schöngart et al 2002, 2005), which at this site is approximately from May to November, largely coinciding with the dry season in most of the Amazon (Online Resource SIFig. 1).

Cores were taken from the hyperdominant tree species *M. acaciifolium* (ter Steege et al. 2013), a brevi-deciduous species, *i.e.* trees which shed their leaves straight after flood levels rise and produce new leaves few days after (Schöngart et al. 2002). Tree-rings of this species have been shown to be annual both by standard dendrochronology and by radiocarbon analysis (Schöngart et al. 2005; Assahira et al. 2017; Batista and Schöngart 2018).

### Isotope analysis

We analysed the isotopic ratio of oxygen in the cellulose extracted from wood cross-sectional laths (Kagawa et al. 2015), using the chemical treatment described in Wieloch et al (2011). The cellulose from individual rings was analysed by pyrolysis over glassy carbon at 1400 °C and an Isotope Ratio Mass Spectrometer (Sercon 20-20 IRMS) in the Environmental Stable Isotope Laboratory of the University of Leicester. All isotopic ratios are expressed relative to the Vienna Standard Mean Ocean Water, in ‰ units.

### Chronology building and dating

We first assessed the strength of isotopic signals from different tree ring sections (i.e., first, middle and last 1/3 section) for a sequence of 20 rings from three different cores. This initial analysis revealed that δ^18^O_TR_ in the middle ring section had the strongest and most significant correlation with basin-wide large-scale precipitation and Amazon River levels (Cintra et al. 2019). We suspect this particular section corresponds most strongly to (upstream) precipitation, as it corresponds to the middle of the growing season when river levels are at their lowest, and trees thus most likely only use precipitation water for growth. We then analysed the δ^18^O_TR_ from the middle ring sections of six different trees for approximately 50–90 rings per tree, producing a record that dates back to 1925 (Online Resource SIFig. 2).

The inter-annual variation of the δ^18^O from the six individual series agreed well, as indicated by the average of all correlations between each series (*r* = 0.58) and the expressed population signal (EPS = 0.89) for the period from 1970 to 2014 (Online Resource SIFig. 2). Estimated years of tree ring formation after cross-dating closely matched the radiocarbon dates of the cellulose in the tree rings (Online Resource SIFig. 3), with few deviations of no more than 2 years and only for dates before 1970 C.E. These analyses confirm that our samples are dated with higher confidence for dates after 1970 C.E. allowing us to perform correlations with climate variables.

### Data analysis

We used Pearson correlations to evaluate the association of the δ^18^O_TR_ series with rainfall, temperature and outgoing longwave radiation (OLR) at local and Amazon-wide scales. OLR was used as it is frequently interpreted as an indicator of convective activity and rainfall (Kousky 1988; Liebmann et al. 1998; Garcia and Kayano 2009). As a separate measure of large-scale hydrological variation, we also correlated δ^18^O_TR_ with levels of Amazonian rivers that drain rainfall over large sub-catchments (Brienen et al. 2012; Gloor et al. 2013). Influences of large-scale climate on δ^18^O_TR_ and teleconnections with sea surface temperatures (SSTs) were visualized using spatial correlation maps of the δ^18^O_TR_ records with gridded precipitation and temperature from the Climate Research Unit climatology (CRUTS 4.00, Harris et al 2020), OLR from NOAA (Lee et al. 2007), and SST from HADISST (Rayner et al. 2003). For comparison, we also used the ERA5 precipitation and temperature reanalysis from ECMWF (Copernicus Climate Change Service, Hersbach et al. 2013). All correlations were calculated for periods post 1970, which is the period with higher confidence in the climate records and for which the δ^18^O_TR_ record is well replicated and precisely dated.

To determine the possible region of δ^18^O rainout during air parcel transport to the study site, we calculated 10-day back trajectories of airmass using a Lagrangian air mass trajectory model (c.f. Baker et al 2016) starting at five heights above the surface (0.99, 0.90, 0.80, 0.70 and 0.60 hPa) for the period of 2000 to 2014 C.E (Fig. 1e). We accumulated precipitation along all airmass trajectories during the time spent over land for days where precipitation at the site was greater than 0 mm. For this analysis we used daily precipitation estimates from the Tropical Rainfall Measuring Mission (TRMM 3B42, GES DISC 2016) (Huffman et al. 2007). We then calculated 3-monthly averaged cumulated precipitation along all trajectories per year and compared the obtained time series with our δ^18^O_TR_ record.

To assess the robustness of the associations between the δ^18^O_TR_ and inter-annual variation in climate, we repeated the δ^18^O—climate analyses using two alternative δ^18^O_TR_ time series: one series from which we removed the long-term trend using a liner regression, and one series with first-order autocorrelation removed. We note that first-order autocorrelation was relatively weak (*r* = 0.3) and that the raw and autocorrelation-detrended records were nearly identical (Online Resource SIFig. 4) with Pearsons’s correlation coefficient *r* = 0.9 for the entire record and *r* = 0.94 for the period from 1970 to 2014. Results of this analysis are presented in Online Resource SIFigs. 4 and 5.

To aid in the interpretation of trends in the δ^18^O record, we estimated the possible contributions of known processes affecting long-term variation in tree ring δ^18^O through changes in plant source water δ^18^O, leaf water enrichment and isotopic exchange with stem water during cellulose synthesis. We used a Rayleigh distillation model to estimate the effect of changes in large-scale precipitation and associated rainout processes on rainfall δ^18^O. This model is sensitive to changes in rainfall amounts along airmass trajectories and to changes in moisture inflow into the Basin. Considering changes in moisture inflow is important because they indicate how much change in rainfall δ^18^O can be expected even if there are no changes in precipitation along rainfall trajectories. Changes in moisture inflow were estimated as in Baker et al (2016) (Online Resource SIFig. 6a). We then used tree ring isotope models to estimate the possible effects of local climate on leaf water enrichment via leaf transpiration, as in Cintra et al. (2019). These models take into account changes in equilibrium and kinetic fractionation resulting from variations in temperature and vapor pressure deficit, the effects of stomatal conductance (*g*_*s*_) on the diffusion of enriched water through the leaf lamina (i.e. Péclet Effect), and isotopic equilibration with water during cellulose synthesis (Barbour and Farquhar 2000; Barbour 2007; Sternberg 2008).

For the estimates of the contributions of different processes to the trend in the δ^18^O_TR_, we considered possible δ^18^O_TR_ changes resulting from 20% reductions in rainfall during the dry season, as indicated by climate analysis for the period of 1970–2014 (Fu et al 2013; Haghtalab et al 2020; Gloor et al 2013, 2015), and an estimated increase of 15% in moisture inflow for the same period, both of which may affect plant source water δ^18^O. We considered an initial δ^18^O of − 11.5‰ for the vapor inflow, estimated from equilibrium with rainfall δ^18^O (Gat 1996) from Belém/Brazil GNIP station, and − 4.5‰ for rainfall at the sampling site during June-October (Iquitos Station, GNIP). For variables that influence δ^18^O_TR_ via leaf water enrichment, we considered a total 2% increase in VPD since 1970, which is what is observed at our site according to data from CRU TS 4.04 (Online Resource SIFig. 6b). With these tree ring isotope models we also consider how much δ^18^O_TR_ change may be expected if plants have down-regulated their *g*_*s*_ rates in response to increasing concentrations of atmospheric CO_2_. Because there might be a lot of variation of this effect for different species, as a conservative measure we consider what would be the maximum possible reduction in *g*_*s*_. Evidence from sub-fossil leaf material, carbon isotopes in tree rings and free air CO_2_ fertilization experiments (FACE) (Wullschleger et al. 2002; Ainsworth and Rogers 2007; Cernusak et al. 2011, 2013; Lammertsma et al. 2011) suggest possible *g*_*s*_ reductions of up to 30% per 100 ppm increase in atmospheric [CO_2_]. Over the period from 1970 to 2014 C.E., atmospheric CO_2_ has increased approximately by 85 ppm. Thus, we consider here a maximum 25% *g*_*s*_ reduction in response to increases in atmospheric CO_2_ concentrations over this period.

All analyses and graphs were done in R.

## Results

Our correlation analyses showed that δ^18^O_TR_ was mainly associated with large-scale climate variability across the Amazon Basin, and to a lesser extent with local climate variation (Table 1 and Online Resource SIFig. 7). At the large scale (Fig. 1), we found δ^18^O_TR_ was positively associated with Amazon dry season temperature (Jun–Oct *r* = 0.59, *p* < 0.01) and OLR (Jun–Oct, *r* = 0.58, *p* < 0.01), and negatively associated with Amazon dry season precipitation (Jun–Oct, *r* = − 0.57, *p* < 0.01) and Amazon river levels measured at Óbidos during the season with lowest river levels (Aug–Nov, *r* = − 0.47, *p* < 0.01). Discharge at this station drains about 77% of rainfall runoff from the Amazon’s catchment (Callède et al 2004, Fig. 1d). These associations are evident both in the spatial-correlation maps and in the time series comparisons (Fig. 1 and Online Resource SIFig. 7), and were nearly the same when evaluated after removing autocorrelation from the δ^18^O_TR_ record (Online Resource SIFig. 5). Correlations between δ^18^O_TR_ and temperature were due to long-term trends in both records, as local and large-scale correlations between δ^18^O_TR_ and temperature disappeared when removing long-term trends (see Table 1, from *r* = 0.59 and *p* < 0.001 to *r* = 0.27, *p* = 0.07). For all other variables, detrending weakened correlations somewhat, but significant correlations remained (Table 1). We found only minimal effects of detrending on the relationships with Amazon River level and Amazon OLR, suggesting a strong role of large-scale precipitation and OLR on the δ^18^O_TR_ variation at the inter-annual scale (Fig. 1).

**Table 1:** Correlations between the δ^18^O_TR_ record and local and large-scale (Amazon-wide) variations in climate for the period 1970–2014

Climate Variable	Pearson’s *r*	(detrended)	Season (months)
Local temperature	0.40**	(0.24)	Dry (June–October)
Local rainfall	− 0.04	(0.08)	Dry (June–October)
Amazon-wide rainfall	− 0.57***	(− 0.44**)	Dry (June–October)
Amazon River discharge at Obidos	− 0.47*	(− 0.44**)	Low (August–November)
Amazon-wide OLR	0.58***	(0.55***)	Dry (June–October)
Amazon-wide temperature	0.59***	(0.27)	Dry (June–October)
TNA sea surface temperature	0.49***	(0.34*)	(February–June)
Nino3.4 sea surface temperature	0.38*	(0.45**)	(February–June)
Partial correlations			
Amazon-wide rainfall, controlled for local T	− 0.41**		Dry (June–October)
Local Temperature, controlled for Amazon rainfall	0.15		Dry (June–October)

Pearson correlations, or partial correlations for raw and detrended climate and δ^18^O_tr_ are shown. Asterisks indicate the significance levels < 0.05*, < 0.01**, < 0.001***

The dominant role of large-scale rainfall on the inter-annual δ^18^O_TR_ record was also evident from its correlations with accumulated precipitation along airmass trajectories (Fig. 1e, j), showing consistent effects of accumulated precipitation for the first half of the dry season (June–August) for all trajectories at heights above 900 hPa, with maximum correlations observed at 600 hPa (*r* = − 0.8, *p* < 0.001, Fig. 1j). At this height, accumulated precipitation averaged for the entire growing season is also significantly correlated with δ^18^O_TR_ (*r* = − 0.54, *p* < 0.041). The air mass trajectories match the regions of influence of rainfall and OLR on δ^18^O_TR_ (Fig. 1a, c, e).

**Fig. 2:**
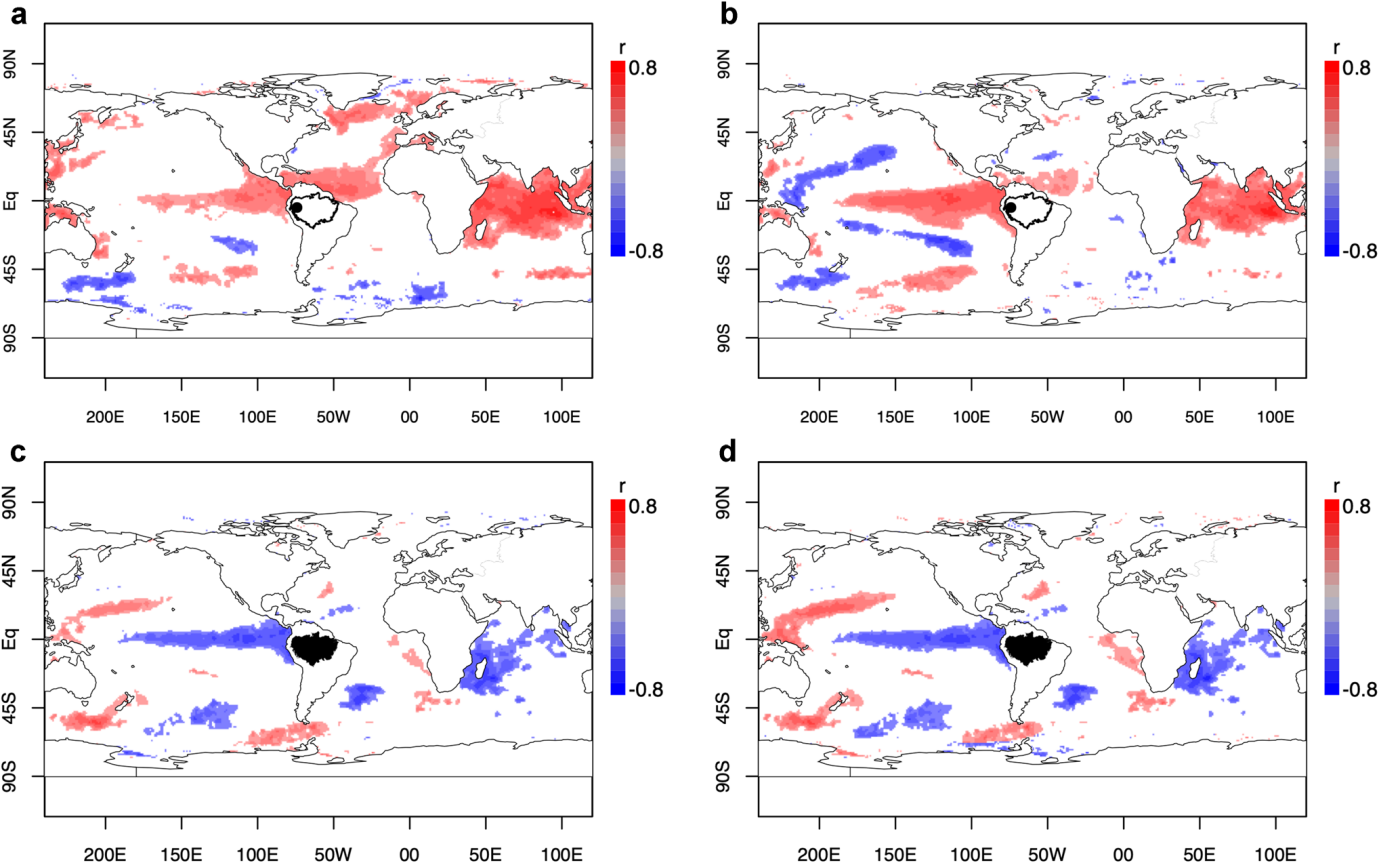
**a** Global spatial correlation maps of δ^18^O_TR_ with sea surface temperatures (SST). **b** as in (**a**) but with long-term trend removed from both SST and δ^18^O_TR_. **c** Global spatial correlation maps of Amazon dry season (Jun-Oct) precipitation with SST. **d** As in (**c**) but with long term trend removed from the data. The black dot in northwest South America indicates the location of the sampling site. Only correlations with *p* < 0.05 are shown

Associations with large-scale hydrology are also reflected in correlations with SSTs globally, showing a strong positive covariation between Tropical North Atlantic SSTs and the raw δ^18^O_TR_ record, and a clear relation with El Niño Southern Oscillation (ENSO), especially at inter-annual time-scales (i.e., the detrended record) (Fig. 2). These regions are known to influence Amazon hydrology (Foley et al. 2002; Yoon and Zeng 2010; Barichivich et al. 2018). We furthermore find a strong positive association with SSTs in the Indian Ocean, which is probably the result of teleconnections of this region with ENSO (Stuecker et al. 2017; Zhang et al. 2021). Associations were strongest with SST averaged over the first half of the year—i.e. during the 6 months preceding the trees’ growing season (Table 1). This time lag in correlations between SSTs and our δ^18^O_TR_ record is similar to the lag observed between Amazon precipitation and SST anomalies (see Fig. 2 and Online Resource SIFigs. 9 and 10) and consistent with other studies (e.g. Yoon and Zeng 2010, their Fig. 4b).

An independent relation of local climate on the δ^18^O_TR_ record was evident in a positive correlation with local dry season temperature from July to September (*r* = 0.43, *p* < 0.05, see Online Resource SIFig. 7c), possibly arising by temperature affecting leaf water enrichment. Thus, we investigated the contribution of each of these variables on variation in δ^18^O_TR_ using partial linear regression analysis (Table 1). In this analysis, the effect of a second climate variable (*z*) on δ^18^O_TR_ was removed by using the residuals of the regression between this control variable *z* and δ^18^O_TR_ to test for the relationship between δ^18^O_TR_ and climate variable *y*. After removing δ^18^O_TR_ variations associated with local temperature, the strength of the correlations with large-scale precipitation were reduced, but remained significant (from *r* = − 0.57, *p* < 0.001 to *r* = − 0.41, *p* < 0.01). In contrast, the correlation of the δ^18^O_TR_ variation with local temperature disappeared entirely (from *r* = 0.43, *p* < 0.05 to *r* = 0.15, *p* = 0.32) after removing the δ^18^O_TR_ variation associated with large-scale precipitation. These results indicate that local temperature has little direct effect on δ^18^O_TR_ at our site and thus that leaf water enrichment does not strongly affect inter-annual variation in δ^18^O_TR_.

**Fig. 3:**
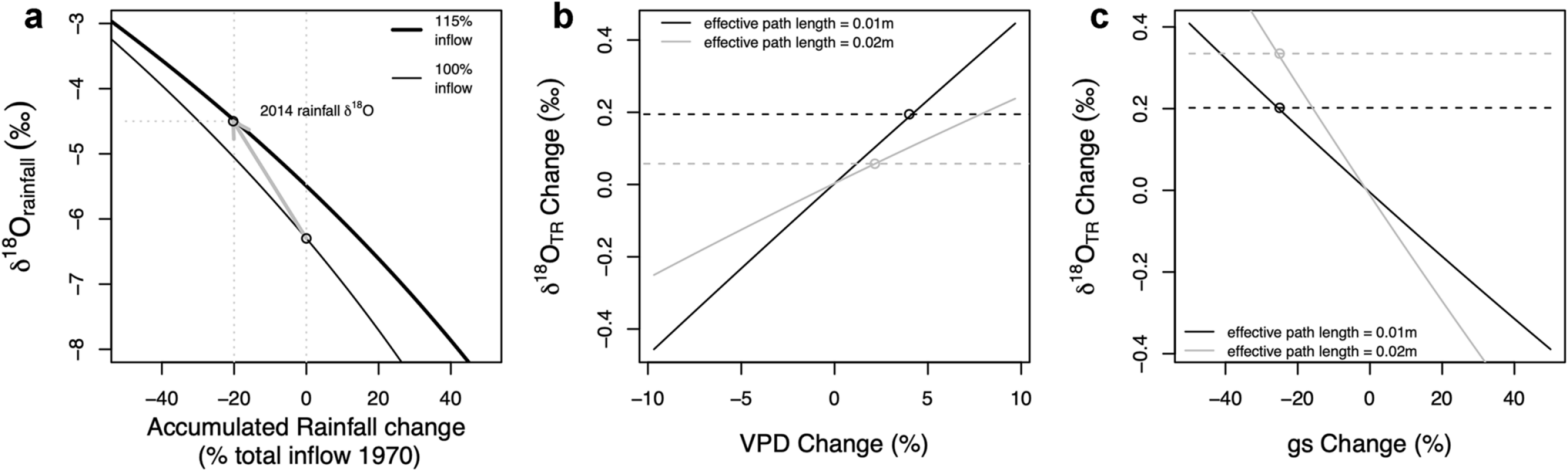
Predicted changes in δ^18^O_TR_ from **a** changes in moisture inflow and large-scale rainfall amount, **b** change in vapor pressure deficit, and **c** reductions in stomatal conductance (*g*_*s*_). Panel (**a**) shows the predicted relationship between δ^18^O_rainfall_ and accumulated rainfall relative to the total moisture inflow in 1970 (%) using a Rayleigh model. The thin line simulates a baseline Rayleigh model (i.e. starting with 100% moisture inflow), while the thick line simulates a model with 15% greater inflow to replicate the observed and modelled increase in inflow and modelled changes in δ^18^O from 1970 and 2014, indicated by the gray arrow (see Online Resource SIFig. 6). The value of − 4.5‰ is the δ^18^O of “dry season” rainfall at the sampling site. Note that accumulated rainfall amounts also account for 50% recycled rainfall, so the value of 1 corresponds to approximately 50% fractional rainout for 1970, and 44% for 2014. Expectations in (**b**, **c**) were based on tree-ring isotopes models (e.g. Cintra et al. 2019), with the black and gray lines indicating a path length of 0.01 and 0.02, respectively. See “Methods” section for details on the models used

The δ^18^O_TR_ record shows significant long-term variation (*p* < 0.01, *r*^2^ = 0.31), with a ~ 2‰ increase in δ^18^O_TR_ since 1970 (Online Resource SIFig. 2). This trend was still highly significant (*p* < 0.01) after removing autocorrelation from the data (Online Resource SIFig. 4). We here use the Rayleigh and tree-ring isotope models to estimate the relative influence of changes in precipitation and moisture inflow, VPD and possible change in stomatal conductance on δ^18^O_TR_. We estimate that a 20% decrease in large-scale rainfall, with a simultaneous 15% increase in moisture inflow, would lead to nearly 2‰ increase in the δ^18^O_TR_ record (Fig. 3a). In contrast, VPD and *g*_*s*_ have much smaller effects on tree ring δ^18^O. A decrease in VPD in the order of 2%, similar to observed changes at the study site, would lead to less than 0.06–0.2‰ change in δ^18^O_TR_ (Fig. 3b). Furthermore, a reduction in *g*_*s*_ of 25% (the maximum realistic change over the study period, see Data Analysis in the “Methods” Section) would lead to additional increases of 0.2–0.33‰ in δ^18^O_TR_ (Fig. 3c).

## Discussion

We produced a δ^18^O_TR_ record based on *M. acaciifolium* floodplain trees from one of the wettest locations in the Amazon to test if it was suitable to record past dry season precipitation variability, and to explore whether the Amazon dry season has changed over recent decades.

The primary source of variation of δ^18^O_TR_ is the plant’s source water δ^18^O, which for most trees comes from recent precipitation. For large catchments such as the Amazon basin, variation in precipitation δ^18^O is the result of a Rayleigh distillation type rainout processes, and thus reflects precipitation amount along the airmass trajectories (Salati et al 1979; Vimeux et al 2005; Vuille and Werner 2005; Ampuero et al 2020; Baker et al 2016; Hurley et al 2018) travelling from the Atlantic coast to the site. Our results show clear evidence of associations between the δ^18^O_TR_ record and various metrics that can be related to large-scale rainout, including basin-wide precipitation, OLR, and Amazon River level, as well as strong correlations with accumulated rainfall along airmass trajectories (Fig. 1). The record shows strongest correlations within the main growing season of these floodplain trees from June to October. During this period, precipitation at our study site is still relatively high, exceeding 150 mm mo^−1^ (see Online Resource SIFig. 1), but it drops to less than 100 mm mo^−1^ over large parts of the Amazon Basin, and many regions receive even less than 50 mm for some of the months during this period.

Besides the expected influence of precipitation on δ^18^O_TR_, we also found an extensive area of influence of surface temperature on δ^18^O_TR_ (Fig. 1b). As temperature and precipitation are strongly anticorrelated, especially at large-scale due co-variation with ENSO (Jiménez-Muñoz et al. 2013, 2016), the effects of large-scale temperature and rainfall could be confounded. While it is hard to disentangle the individual climate effects on δ^18^O_TR_, several arguments support the notion that large-scale rainfall is the major—or sole—driver of the δ^18^O_TR_ variations. Firstly, while precipitation amount necessarily affects rainfall δ^18^O through upwind rainout (ie. Rayleigh distillation), temperature is not expected to be an important driver of rainfall δ^18^O in the tropics (Dansgaard 1964; Vuille and Werner 2005; Risi et al. 2008; Baker et al. 2016; Ampuero et al. 2020). Indeed, modelling studies (e.g. Vuille and Werner 2005), and records of tree ring δ^18^O from the Amazon basin during the wet season (Baker et al. 2016), Andean ice core δ^18^O (Hurley et al. 2018), western Amazon speleothem δ^18^O (Kanner et al. 2013) and δ^18^O varve records (Bird et al. 2011), all indicate that Amazon convection is the main control on variation in precipitation δ^18^O, and not temperature. Moreover, although the correlations with both large-scale rainfall and large-scale temperature remain significant even when tested with partial regressions, the association between δ^18^O_TR_ and large-scale temperature is highly dependent on the long-term trend in both the δ^18^O_TR_ record and the climate data, and completely disappears once the trend is removed (Fig. 1e, Fig. 2f). Thus, it is unlikely that the δ^18^O_TR_ record reflects a direct influence of large-scale temperature on rainfall δ^18^O, and this association may simply arise as an artefact of the large-scale external controls on climate conditions, such as ENSO, which results in strong co-variations between precipitation and temperature.

These results thus suggest that our record mainly reflects the rainout of heavy isotopes within the basin (Dansgaard 1964; Salati et al. 1979), i.e. the gradual removal of heavy water along moisture trajectories (Rayleigh distillation), which leaves an imprint on the δ^18^O of rainfall water that the trees take up from the soil. Uptake of river water left in the soil is also possible in the beginning of the growth period, but unlikely to be reflected in our δ^18^O_TR_. This is because our record was produced using only the middle portion of the tree rings, which correspond to wood formed during the main growing season, when river levels may drop several meters below the surface (Schöngart et al. 2002; Cintra et al. 2019). At this stage, river or deeper ground water pools would probably be out of reach and/or inaccessible for these trees, especially when the local climate conditions provide enough rain to maintain wet soils (Bertrand et al. 2014; Evaristo et al. 2015; Barbeta and Peñuelas 2017). The lack of any signal from previous seasons (Online Resource SIFig. 7) confirms that the δ^18^O_TR_ is unlikely to be influenced by river water uptake, because river water should carry the δ^18^O of rainfall runoff from previous months. Thus, we find it most likely that the δ^18^O_TR_ signals originate from the δ^18^O of rainfall during the main growth season of the trees.

It is remarkable that the δ^18^O_TR_ of the floodplain trees used in this study mainly record a source water δ^18^O signal. Previous research on this species showed that local climate can exert an effect on the tree ring δ^18^O due to leaf water enrichment during leaf transpiration (e.g. the Péclet Effect). These effects are however expected to be greater at drier sites compared to more humid sites (Cintra et al. 2019). We thus suspect that the rather wet conditions year-round at this site (Online Resource SIFig. 1a) may limit leaf water enrichment variations, which might otherwise weaken climate signals from source water δ^18^O (Cintra et al. 2019). This is probably the reason why we did not find consistent correlations with local climate variations. In summary, our analysis indicates that these floodplain trees record variation in precipitation δ^18^O during the growing periods for these trees, which corresponds to the driest period of the Amazon basin upstream of the study site. These results suggest the δ^18^O_TR_ record presented here may provide a proxy for Amazon dry season precipitation amount.

### Decadal climate changes inferred from the δ^18^O_TR_ record

An outstanding feature of the floodplain δ^18^O_TR_ record is a decadal-scale upward trend of 2‰ from 1970 to 2014. If this trend truthfully reflects changes in rainfall δ^18^O, it would suggest large-scale rainfall variations during the dry season in the Amazon. We are particularly interested in the increasing δ^18^O_TR_ values over the period from 1970 to 2014 (Fig. 2), because this period coincides with the start of an intensification of the hydrological cycle in the Amazon Basin (Gloor et al. 2013; Barichivich et al. 2018), and because this is the most well-replicated segment of the record, for which we have the highest dating confidence. We thus considered how much rainfall change can be inferred from this δ^18^O_TR_ trend, based solely on changes in rainfall δ^18^O according to a Rayleigh rainout framework.

By simply considering the slope of the linear relationship between inter-annual variation of δ^18^O_TR_ and large scale rainfall we infer a dry-season rainfall reduction of ~ 50 mm over the period from 1970 to 2014, which is equivalent to a 30% reduction in rainfall upstream of the study site during June to October, the Amazon “dry” season. For comparison, a pure Rayleigh distillation model would indicate that nearly 2‰ increase in rainfall δ^18^O could result from ~ 20% reduction in accumulated rainfall along airmass trajectories (Fig. 3a), taking into account changes in moisture inflow into the Basin (Online Resource SIFig. 6a). This estimate is in fair agreement with observations of decreasing rainfall during the dry season in this region (Fu et al. 2013; Haghtalab et al. 2020), which indicate small reductions of up to 20 mm (nearly 20%) per month during the 3 driest months since 1990 (Gloor et al. 2013, 2015). These trends are observed for most of the Amazon region except the north-western portion of the Basin, consistent with what we observe here (Fig. 1). Whether this trend of intensification of the dry season will persist into the future is still uncertain (Boisier et al. 2015). From the decadal-scale fluctuations in our record, we may infer that the current climate conditions during the Amazon dry season are not unprecedented, as the δ^18^O_TR_ record shows a first peak around 1940. This suggests that the observed trends in our δ^18^O_TR_ record may result at least partially from long-term natural climate cycles. We note that this should be interpreted with caution as for this period, the record may not be dated with absolute precision and is not well replicated. Nevertheless, this would be in line with another 259-year long tree ring width record, which shows a multidecadal pattern of variation in rainfall amounts with a frequency of 35 years (Granato-Souza et al. 2020).

Multidecadal climate fluctuations in the Amazon Basin are driven by fluctuations of SSTs in adjacent ocean basins (Yoon and Zeng 2010). Indeed, our δ^18^O_TR_ record shows a clear connection with SST anomalies in the surrounding oceans, that closely resembles the effect of SST anomalies on Amazon dry season rainfall (Fig. 2). In particular, SSTs in the Tropical North Atlantic (TNA) Ocean have been warming since approximately 1970, which coincides with the start of the trend that we see in our δ^18^O_TR_ record. The TNA Ocean is source region for Amazon moisture and one of the main drivers of variations in moisture inflow and thus dry season rainfall amounts for large areas of the Basin (Yoon and Zeng 2010). Warming of the TNA Ocean has previously been associated with reductions in dry season rainfall in the Amazon, because it may change the trade winds that bring moisture into the Amazon to a more northern position. Thus, the effect the long-term warming of TNA Ocean SSTs on Amazon dry season rainfall may possibly be one cause to the trend we observe in the δ^18^O_TR_ record. These connections support the possibility that the 1970–2014 trend in the δ^18^O_TR_ may reflect decadal-scale reductions in dry season rainfall, associated with SST controls on large-scale circulation patterns.

Interestingly, the multidecadal fluctuations in our δ^18^O_TR_ record for the period of 1925–2014 (Online Resource SIFig. 2) do not differ much from the fluctuations in the Atlantic Multidecadal Oscillation (Online Resource SIFig. 6c) (Kerr 2000; Enfield et al. 2001). While this might be coincidental, the AMO is expected to be reflected in the Tropical North Atlantic SST (Kerr 2000; Enfield et al. 2001; Barichivich et al. 2018). Recent studies suggest that global warming may have aggravated the recent warming of SSTs in Northern Atlantic Ocean (Biastoch and Böning 2013; Biastoch et al. 2015; Barichivich et al. 2018), and its reflection in the TNA SSTs may be one of the causes for much of the ongoing climate changes in the Amazon (Gloor et al. 2013, 2015; Barichivich et al. 2018; Wang et al. 2018).

As our record is relatively short, we cannot decisively conclude if the drying trend is a result of natural climate variability only, or if it has been aggravated by anthropogenic climate change. If anthropogenic climate change has contributed to this trend, then intensification of the dry season could persist into the future. This would be in line with CMIP5 model predictions for the next century (Kitoh et al. 2013; Fernandes et al. 2015; Li et al. 2016; Hua et al. 2019), and could have severe consequences for the region with impacts on the local socioeconomic sectors and livelihoods (Marengo et al. 2018), the forest’s carbon balance (Quesada et al. 2012; Johnson et al. 2016) and biodiversity (Esquivel-Muelbert et al. 2017, 2018), and hamper forest regeneration in deforested areas.

Given the far-reaching implications of the long-term drying trend implied by our record for current understanding of climate changes in the Amazon, we also consider whether additional factors may have contributed to the observed trend in the δ^18^O_TR_ time-series. A first consideration is that observed long-term increases of approximately 1.0 °C in large-scale temperature may have affected the equilibrium fractionation during the formation of raindrops. However, according to known temperature effects on fractionation processes (Botinga and Craig 1969), this effect would result in only ~ 0.1 ‰ change in rainfall δ^18^O. Another hypothesis is that changes in total evapotranspiration from the forest may affect the δ^18^O_TR_ record. Forests play an important role by recycling precipitation through evapotranspiration, which contributes to the total amount of precipitation downwind. Removal of forest cover caused by large-scale deforestation could lead to reductions in total evapotranspiration and decreased precipitation amounts, predominantly during the dry season (Spracklen et al. 2012; Spracklen and Garcia-Carreras 2015; Khanna et al. 2017; Pattnayak et al. 2019). While we cannot reject the possibility of deforestation as a possible cause for rainfall reductions during the dry season, changes in evapotranspiration due to deforestation may hardly be reflected in rainfall δ^18^O (Pattnayak et al. 2019; Ampuero et al. 2020). Moreover, observations suggest no clear indication of a long-term trend in evapotranspiration during the dry season (Hurley et al. 2015; Zhang et al. 2016; Moura et al. 2019; Sun et al. 2019; Baker et al. 2020, 2021). Thus, the trend in our δ^18^O_TR_ record is likely not driven by large-scale changes in evapotranspiration.

Lastly, we also consider the extent to which the decadal variation in the mean δ^18^O_TR_ might be driven by slower, gradual changes in the degree of leaf water enrichment—even if its effects on the inter-annual scale are negligible. For example, gradual increases in VPD could lead to an increasing leaf water enrichment. Further, down-regulations of stomatal conductance (*g*_*s*_) in response to increasing atmospheric CO_2_ concentrations (Morison 1985; Franks 2013) could affect transpiration, and thus leaf water enrichment (Cooper and Norby 1994). It is hard to assess to what degree this effect has indeed contributed to the trends in the δ^18^O_TR_ record, as *g*_*s*_ responses to CO_2_ vary widely among different species (Lammertsma et al 2011; Cernusak et al 2011; Rahman et al 2020; van der Sleen et al 2015). Nonetheless, we assessed this possible contribution based on the tree-ring models detailed in Cintra et al (2019)—see details in “Methods” Section—data analyses. Our estimate of the effects of VPD reductions at the sampling site together with possible *g*_*s*_ reductions in response to increases in atmospheric CO_2_ over 1970–2014 result in a maximum increase in δ^18^O_TR_ of up to 0.5‰. Furthermore, as VPD has changed very little at our site, no changes in source water δ^18^O are expected from evaporative enrichment of top soil water. Thus, these effect can only partially explain the observed 2‰ δ^18^O_TR_ trend, and only by assuming a large *g*_*s*_ response (i.e., 25% decrease) of these trees to CO_2-_fertilization, which is not known at the moment (see Data Analysis in the “Methods” section for details).

In all, our analyses suggest it is unlikely that the observed long-term trend in the δ^18^O_TR_ record can be explained by changes in widespread forest evapotranspiration or by leaf water enrichment related to long-term increases in local dryness or atmospheric CO_2_ growth. It is more likely that the trend in the δ^18^O_TR_ record primarily reflects changes in plant source water δ^18^O driven by large-scale rainfall reductions during the dry season in the Amazon Basin since approximately 1970, in agreement with climate observations for the region.

## Summary and conclusions

We analysed interannual and long-term variations of oxygen isotope ratios in the tree rings of *M. acaciifolium* trees from a floodplain site located in the western Amazon. We expected this δ^18^O_TR_ would reflect large- scale climate signals imprinted in the plant’s source water δ^18^O via the rainout of heavy isotopes over moisture transport within the basin. As the trees we analysed grow when river flood levels are low, which largely coincides with the Amazon-wide dry season, we also expected that the observed climate signals would correspond to the period of the dry season. As expected, the presented δ^18^O_TR_ record was associated with Amazon-wide hydro-climatic conditions during the dry season. To our knowledge, this is the first published δ^18^O_TR_ record to reflect past dry season hydroclimate variation in the Amazon, complementing previous δ^18^O_TR_ records from the Amazon which reflect wet season climate conditions. The δ^18^O_TR_ record presented here was mainly negatively associated with large-scale rainfall upwind from the sampling site, with little or no influence of local climate. One of the most distinctive features of the record is a multidecadal increase of up to 2‰ over the last 40 years. Our analyses suggest that this most likely reflects a widespread drying trend during the dry season in the Amazon, which is consistent with current observational studies, and thus deserves further attention. Floodplain trees may achieve ages of up to 400 years (Schöngart et al. 2004, 2005; Resende et al. 2020), which may allow us to extend our knowledge of dry season climate fluctuations further back in time.

## Supplementary Information

Below is the link to the electronic supplementary material.

Supplementary file1 (PDF 2240 kb)

## Declarations

### Data availability

The datasets generated during and/or analysed during the current study are available from the corresponding author on reasonable request.

### Code availability

The codes used to analyze the data for this study are available from the corresponding author on reasonable request.
