# Supplementary material for: Tree-ring oxygen isotopes record a decrease in Amazon dry season rainfall over the past 40 years
Source: Clim Dyn. 2021 Nov 26;59(5-6):1401–14. doi: 10.1007/s00382-021-06046-7 (PMC9372001; doi:10.1007/s00382-021-06046-7)
Supplement: Supplementary file 1 — Supplementary file1 (PDF 2240 kb) [file 382_2021_6046_MOESM1_ESM.pdf]

Supplementary material for the research article " Tree-ring oxygen isotopes record a decrease in Amazon dry season rainfall over the past 40 years"

Bruno B L Cintra[1,2], Manuel Gloor[1], Arnoud Boom[3], Jochen Schöngart[4], Jessica C. A. Baker[5], Francisco W Cruz[6], Santiago Clerici[1], Roel Brienen[1].

1. School of Geography, University of Leeds, Garstang North Building, LS2 9JT, Leeds, United Kingdom;
2. Institute of Biosciences, University of São Paulo, Rua do Matão 14, 05508-090, São Paulo, Brazil;
3. School of Geography, Geology and the Environment, University of Leicester, Bennet Building, University Road, Leicester, LE1 7RH, United Kingdom;
4. Coordination of Environmental Dynamics, National Institute for Amazon Research, Av. André Araújo 2936, Petrópolis, Manaus 69067-375, Brazil;
5. School of Earth and Environment, University of Leeds, Leeds, United Kingdom;
6. Institute of Geosciences, University of São Paulo, Rua do Lago 562, 05508-080, São Paulo, Brazil.

Corresponding author: Bruno B L Cintra (brunoblcintra@gmail.com)

Supplementary Figures

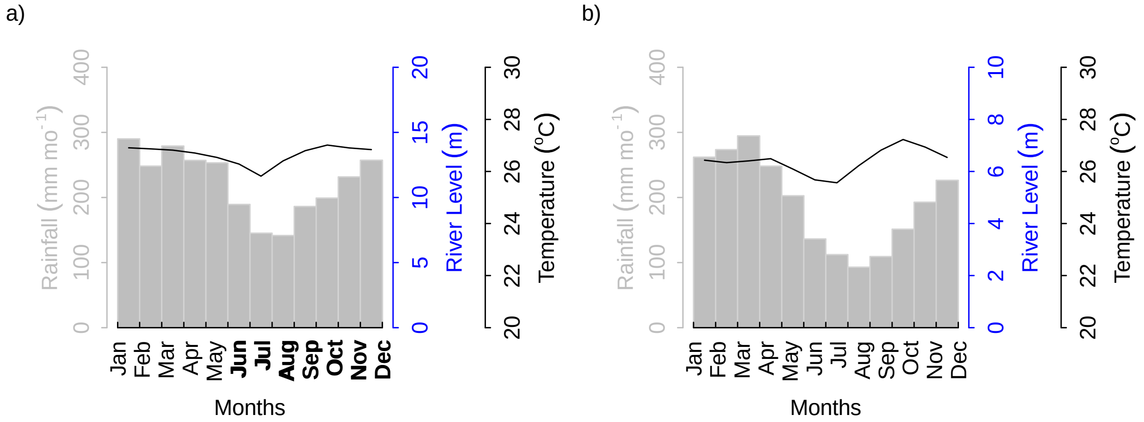

Supplementary Fig. 1 Seasonal variations in rainfall, river levels and temperature in (a) the sampling site and (b) Amazon Basin. River data in (b) are from the Obidos river gauge station from Brazilian National Waters Agency

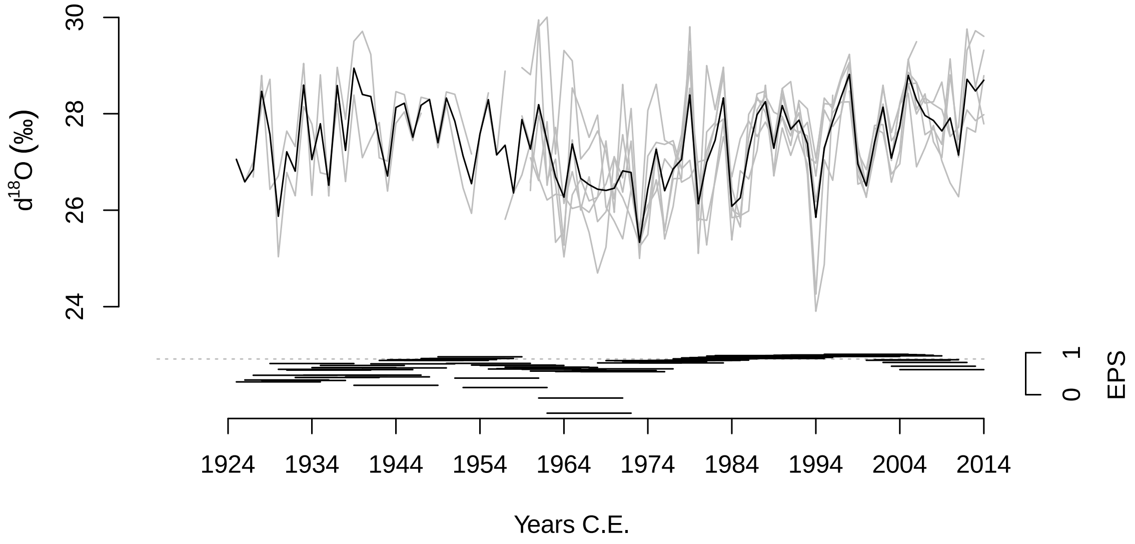

Supplementary Fig. 2 $\delta^{18}O_{TR}$ record based on six *Macrolobium acaciifolium* trees from western Amazon floodplains within the Marañon catchment, Peru, showing 10-year running Expressed Population Signal values

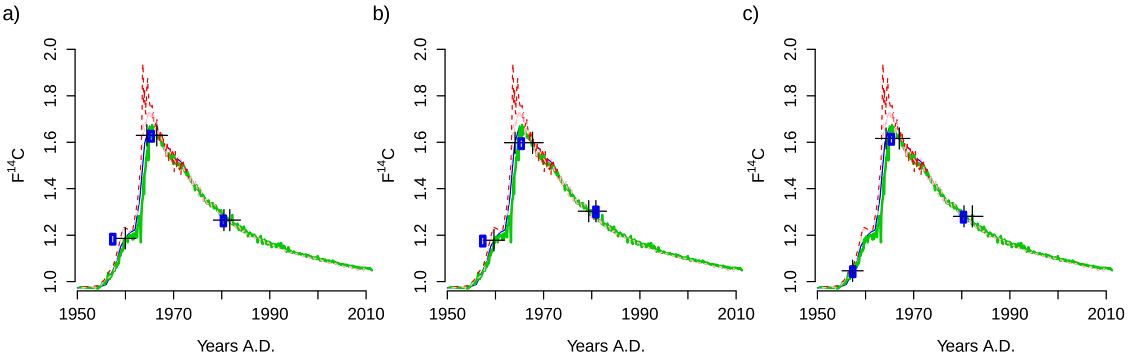

Supplementary Fig. 3 Results from the radiocarbon dating of 9 tree rings from three of the trees used in this study. Each panel shows samples from one tree. Blue circles indicate the ring-dates obtained through wood anatomical analysis and tree-ring cross dating, plotted against their respective measured $F^{14}C$, and back crosses indicate possible radiocarbon dates of the tree rings based on their measured $F^{14}C$ and on the $F^{14}C$ bomb-peak curves from the southern Hemisphere Zones 1-2

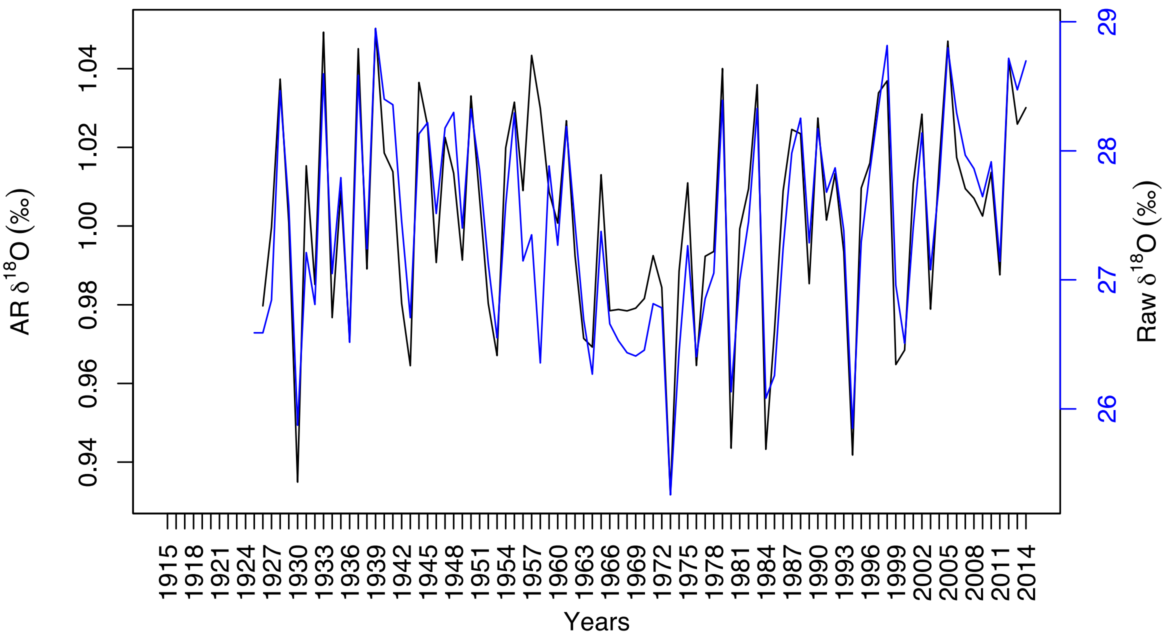

Supplementary Fig 4 Comparison between the raw $\delta^{18}O_{TR}$ record (blue) and the autocorrelation-detrended $\delta^{18}O_{TR}$ (ARδ^{18}O, black). Both series are shown for the full period they span, from 1926 to 2014. The two records are highly correlated (r=0.9), especially for the most recent period from 1970 to 2014 (r=0.94), which corresponds to the most well-dated period of the record.

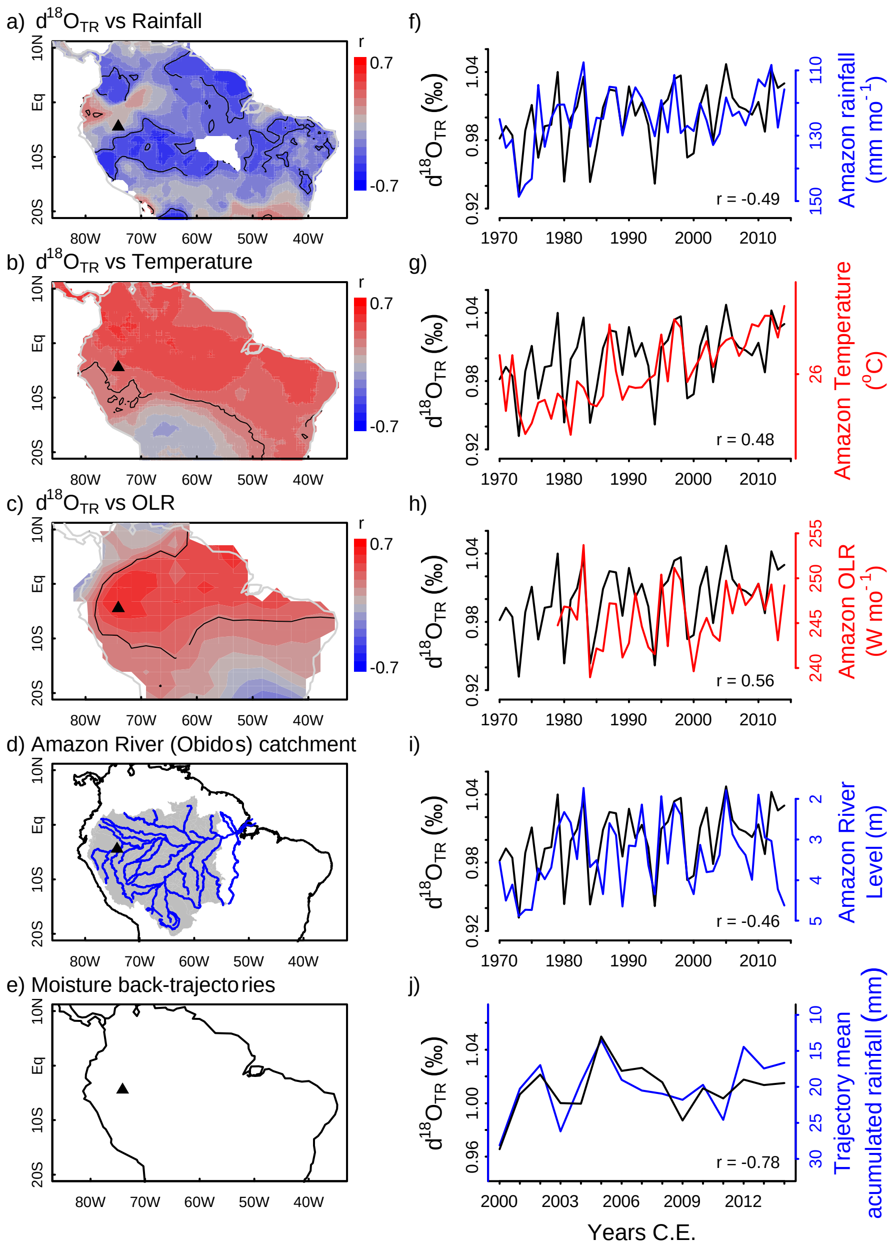

Supplementary Figure 5 Same as Figure 1, but after removing autocorrelation from the $\delta^{18}O_{TR}$ record.

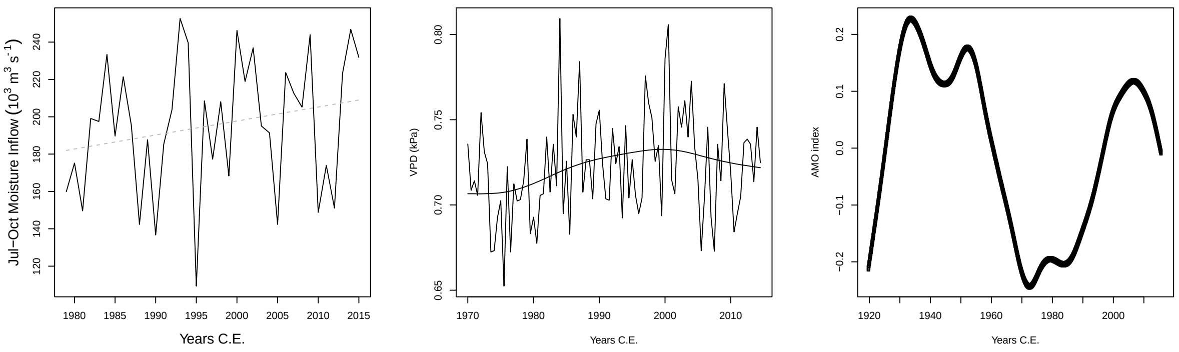

Supplementary Fig. 6 (a) Estimated changes in moisture inflow during the 3 driest months in the Amazon (c.f. Baker et al. 2016) – data only available from 1980 onwards. (b) Change in vapor pressure deficit at the sampling site since 1980. (c) Decadal trend of the Atlantic Multidecadal Oscillation Index since 1920

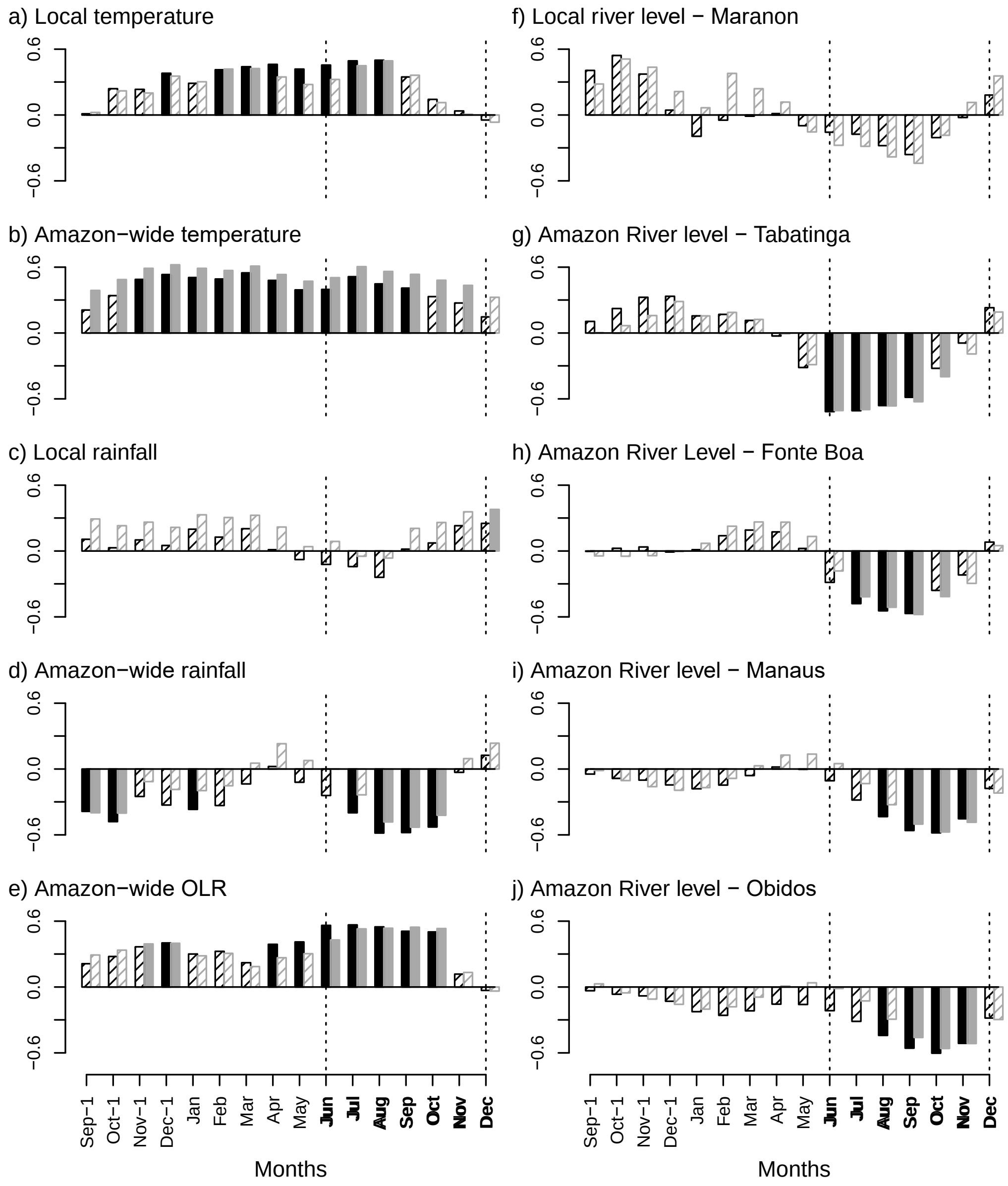

Supplementary Fig. 7 Correlation of the $\delta^{18}O_{TR}$ record with 3-monthly means of environmental variables at local and large scales. Shadings indicate the seasonality (not to scale) of the variable in each graph. The start and end of growing season are indicated by the dotted lines. The growing season is also indicated by the months in bold in the x-axis. Each month correspond to the middle of 3 month averages. Solid bars indicate significant correlations using the observed data (gray) and with the long-term trend removed (black)

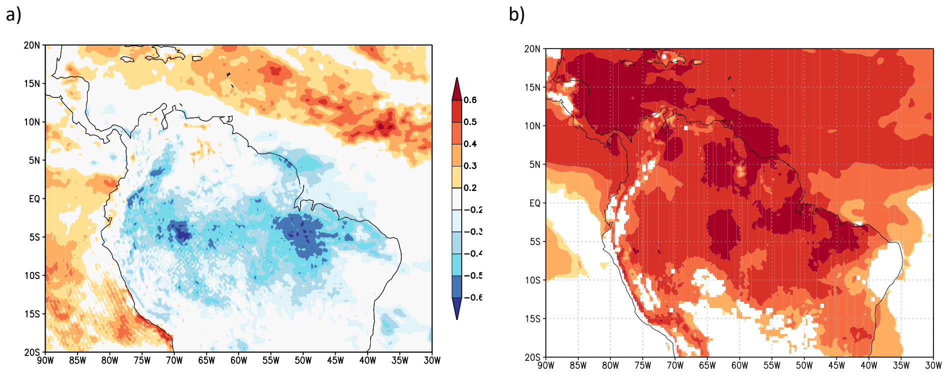

Supplementary Fig. 8 Spatial correlation map of the $\delta^{18}O_{TR}$ with precipitation (a) and temperature (b) from ERA5 reanalysis

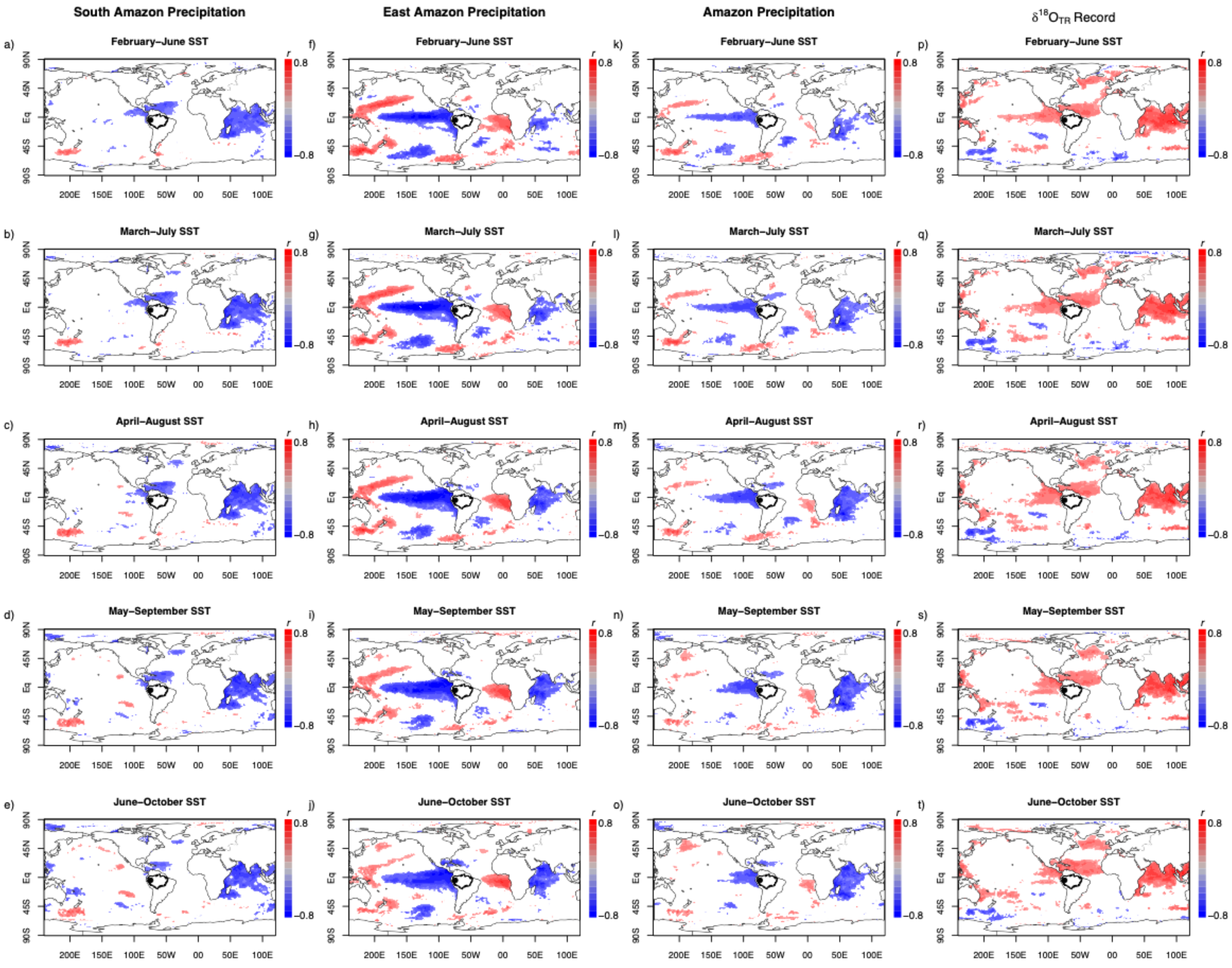

Supplementary Fig. 9 Comparison of spatial correlation patterns of SST with of dry season (June-October) Amazon precipitation and $\delta^{18}O_{TR}$ record from West Amazon floodplain trees which grow during the dry season. (a-e) South Amazon (Amazon basin south of -03° latitude), (f-j) East Amazon (Amazon Basin east of ° longitude), (k-o) entire Amazon Basin, (p-t) $\delta^{18}O_{TR}$ record.

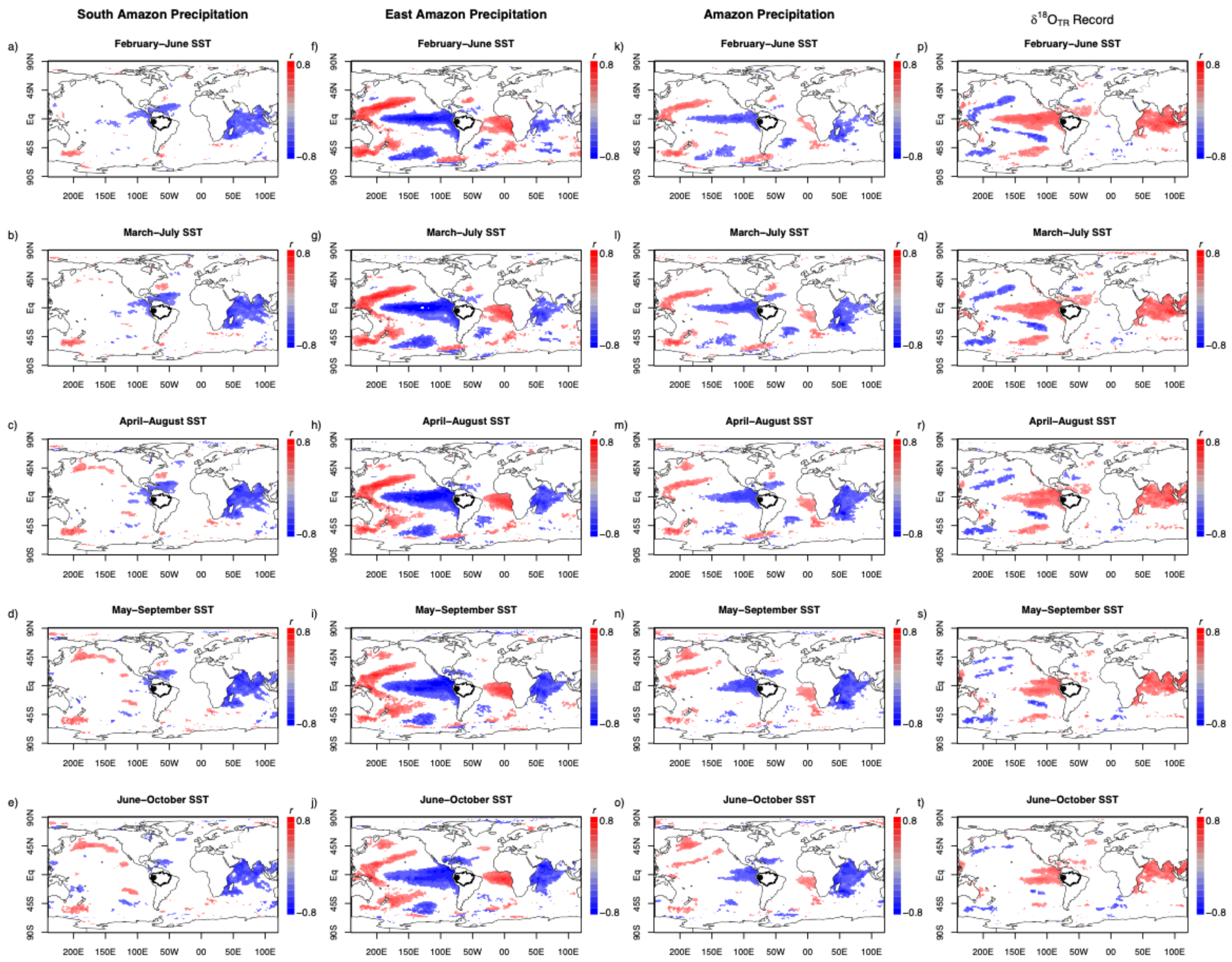

Supplementary Fig. 10 Same as in R1, but after removing the long-term linear trend from the data.
